# Adapting Community Health Worker Care Models to Advance Mental Health Services Among LGBTQ Youth

**DOI:** 10.1007/s10488-023-01268-9

**Published:** 2023-04-18

**Authors:** Miya L. Barnett, Hanan Salem, Yessica Green Rosas, Emily Feinberg, Rocio Nunez-Pepen, Andrea Chu, Hana Belmont-Ryu, Em Matsuno, Sarabeth Broder-Fingert

**Affiliations:** 1 Department of Counseling, Clinical, and School Psychology, Gervitz Graduate School of Education, University of California, Santa Barbara, Santa Barbara, CA 93106-9490 USA; 2 Boston University School of Medicine, Boston, MA USA; 3 Boston University School of Public Health, Boston, MA USA; 4 Boston Medical Center, Boston, MA USA; 5 Arizona State University, Tempe, AZ USA; 6 University of Massachusetts Chan Medical School, Worcester, MA USA

**Keywords:** LGBTQ, Mental health, Community health worker, Racial/ethnic minorities, Equitable care

## Abstract

Lesbian, gay, bisexual, transgender, and queer (LGBTQ) youth of color experience high rates of mental health disorders, yet they experience challenges to accessing mental health services. Community health worker (CHW) models of care have potential to promote equitable mental health services among LGBTQ youth. Our aim was to understand how CHW models could be adapted to better support LGBTQ youth of color in accessing mental health services. Semi-structured qualitative interviews were conducted with LGBTQ youth of color (*n* = 16), caregivers of LGBTQ youth (*n* = 11), and CHWs (*n* = 15) in Massachusetts and California. Interviews were coded by 8 members of the research team. A Rapid Qualitative Analysis was conducted to identify themes. Caregivers, youth, and CHWs all endorsed the value of CHW models for this population. They also almost universally suggested multiple adaptations are needed for the model to be effective. Four main categories of themes emerged related to intervention adaptations: (1) **Why** adaptations are needed for LGBTQ youth, (2) **Who** should serve as CHWs providing care, (3) **How** CHWs should be trained, and (4) **What** content needs to be included in the intervention. Broadly, findings suggest the relevance of having CHW models for LGBTQ youth of color to address stigma and discrimination experienced, access to culturally and linguistically relevant services, and the need for caregiver support of LGBTQ youth. CHWs need increased training in these areas.

## Introduction

Significant mental health disparities exist for lesbian, gay, bisexual, transgender, and queer (LGBTQ) youth (Hafeez et al., 2017). While LGBTQ individuals are more than twice as likely as non-LGBTQ individuals to have a mental health disorder (The Trevor Project, 2022), substance use disorder, or die by suicide (Kann et al., 2016; Ream, 2019), they experience greater difficulty in accessing needed mental health services (Schulman & Erickson-Schroth, 2019; Williams et al., 2018). Anti-LGBTQ stigma exacerbates mental health disparities, in that it contributes to psychological distress and impedes access to care (Holt et al., 2023; Rees et al., 2021). Structurally there is an inadequate workforce to provide affirming care. Additionally, youth may fear disclosing their gender-identity or sexual orientation to their caregivers, who often serve as gatekeepers to receiving mental health services (Holt et al., 2023). These disparities are especially profound for LGBTQ Black, Latinx, Native American and Asian American youth (hereafter referred to as youth of color; Abreu et al., 2022; Hatchel et al., 2021), who experience greater discrimination due to the intersection of multiple minority statuses (Swann et al., 2020). Therefore, service models are needed that can address intersecting experiences of oppression for youth of color and their families.

Indeed, intersectionality, a framework that examines the ways in which interconnected identities, such as race, gender identity, sexuality, and class impact levels of discrimination and inequity individuals encounter, has been shown to be a fitting approach to more effectively examine variations in health inequity across LGBTQ youth (Veenstra, 2011). Evidence suggests that LGBTQ youth of color are at an increased risk of developing depressive symptoms and suicidal thoughts and behaviors compared to White LGBTQ youth (Abreu et al., 2022; Hatchel et al., 2021). These outcomes can vary within LGBTQ youth of color, given how this category embodies different races, ethnicities, gender identities, and sexual orientations. For example, experiencing multiple minority stressors increases the odds of a suicide attempt across all identities for LGBTQ youth; however, American Indian/ Alaskan Native youth were most likely to experience these stressors, which placed them at higher risk for suicide attempts (Green et al., 2022). Findings from a systematic review examining mental health disparities among LGBTQ Latinx youth also found that these intersecting identities were associated with unique challenges impacting their sense of belonging, sense of self, and mental health (Garcia-Perez, 2020). These challenges have been exacerbated by the current social and geopolitical environment that is promoting greater discrimination against groups of color (Castro-Ramirez et al., 2021) and influencing policy changes that threaten LGBTQ individuals’ rights as well as access to services and opportunities (Chappell, 2022; Diaz, 2022). Indeed, one study found that states with higher levels of structural racism and anti-LGBTQ oppression had higher rates drinking and suicide attempts amongst Black sexual minority male youth (English et al., 2022).

Mental health outcomes are better for LGBTQ youth when families are able to support their coming out process; however, parents frequently experience distress and grief related to their child’s sexual and gender identity, impacting their parenting (D’amico et al., 2015; Ryan et al., 2009). A large national study with Latinx, Black, Asian, Native American, and White LGBTQ youth found lower levels of parental support for youth of color in comparison to White youth (Abreu et al., 2022). The authors posited that non-supportive behaviors from caregivers of color might relate to their fears that their child’s intersecting identities could increase their experiences of discrimination and oppression (Abreu et al., 2022). On the other hand, cultural values surrounding family unity (e.g., familismo in Latinx cultures) could be leveraged to promote parental support from caregivers (Abreu et al., 2020). It is critical that LGBTQ youth of color and their families receive services that effectively address how intersecting aspects of their identities impact their mental health. One specific area of focus that is needed is how to bolster parental support for LGTBQ youth of color, to enhance this protective factor for positive mental health outcomes (Abreu et al., 2022).

Partnering with community members with expertise in navigating cultural norms and health care access, such as community health workers (CHWs), is one promising strategy to work with families of LGBTQ youth. CHWs have been identified as an important workforce to enhance service equity for communities of color (Barnett et al., 2018b), and specifically engage caregivers in mental health services (Barnett et al., 2021a). In general, CHWs are individuals from the communities they serve without formal mental health training, who aim to enhance access to care. CHWs include volunteer networks, such as *promotoras de salud*, who typically work with the Latinx community, and hired CHWs who work within healthcare systems (Barnett et al., 2021a). CHWs frequently work with caregivers (e.g., biological, adoptive, or foster parents; grandparents) to enhance access and engagement in care by addressing logistical barriers, stigma, and enhancing the cultural fit of services (Broder-Fingert et al., 2020; Chakawa, 2022; Davis et al., 2022). In one study, a CHW-implemented model was associated in reduced child mental health stigma among Black and African American caregivers (Chakawa, 2022). As such, CHW could be an especially valuable group to work with the family system of LGBTQ youth of color, to enhance mental health access and parental support. One example of a CHW model is family navigation, which has been shown to enhance equity in access to care (Broder-Fingert et al., 2020). Family navigation is an evidence-based, multi-component intervention that incorporates motivational interviewing, problem-solving strategies, patient education, and care coordination to aid families at high-risk for disparities in gaining timely access to needed services. Traditional navigation models train CHWs to assist families in overcoming system and patient-specific barriers to a defined set of services over a time-limited period (Ali-Faisal et al., 2017; Broder-Fingert et al., 2019, 2020). Over the past few years, CHWs have gained prominence as an important answer to some of the US healthcare system’s most pressing problems, with hundreds of millions of dollars being invested in building this workforce (Barnett et al., 2021b). With the rapidly growing interest in this workforce, there is an urgent need for more research on types of CHW models, and how they can be adapted for different populations.

Given that LGBTQ youth of color experience particular challenges with stigma and bias, as well as unique challenges within the family unit (Murphy & Hardaway, 2017), CHWs could be a key workforce to improve access to care. Yet, to date, limited research has identified the roles CHWs may have in working with LGBTQ youth and their caregivers. Social proximity, or having similar cultural and personal backgrounds as the individuals they serve, has been identified as a primary strategy that make CHWs especially adept at helping patients overcome distrust of health systems (Barnett et al., 2021a; Gustafson et al., 2018). However, when thinking of who should be the CHWs serving the families of LGBTQ youth it is important to identify what intersecting traits may be most important for the providers related to culture, gender identity, and sexual orientation.

### Current Study

The purpose of this qualitative study was to identify adaptations needed related to the providers, training, and interventions for CHW models of care to enhance equity for LGBTQ youth of color in accessing mental healthcare. The study design was informed by the tenets of implementation science, which focuses on how to effectively implement evidence-based practices within community settings (Bauer & Kirchner, 2020).One specific area of interest in implementation science is the adaptations that are needed to evidence-based practices to increase the fit for the local context, which can include the individuals served and the providers of the intervention (Stirman et al., 2019). Specifically, the Framework for Reporting Adaptations and Modifications-Expanded (FRAME) was used given its focus on identifying adaptations and modifications of evidence-based practices in a multifaceted and comprehensive manner (Stirman et al., 2019). In this framework, it is recommended to focus on both the process of adaptation and the reasons for modifying interventions. Processes include identifying who determines changes need to occur (e.g., consumers, providers, researchers), the content/nature of changes, and contextual modifications (e.g., who provides the treatment).

This study leveraged ongoing community-academic partnerships with CHWs embedded within healthcare systems in Massachusetts (i.e., family navigators) and community settings in California (i.e., *promotoras*). Qualitative interviews were conducted with LGBTQ youth, caregivers of LGBTQ youth, and CHWs to identify: 1) provider characteristics essential to promoting effective and engaging care, 2) CHW training needs when serving LGBTQ youth, and 3) intervention components that should be incorporated into CHW models such as family navigation.

## Method

### Participants

Inclusion criteria for the study included that participants either identify as (1) currently serving as a CHW, (2) caregiver of a child ages 10–21 who identifies as a sexual or gender minority, or (3) youth between the ages of 18–21. All participants needed to be English or Spanish speaking. Given the focus on making adaptations for LGBTQ youth of color, purposeful sampling was conducted within community partnerships that served racially and ethnically minoritized populations. Additionally, efforts were made to have representation across sexual orientations and gender identities. Participants (*N* = 42) included LGBTQ youth (*n* = 16), caregivers of LGBTQ youth (*n* = 11), and CHWs (*n* = 15) in Massachusetts and California. All caregivers, CHWs, and youth participants reported being racially and/or ethnically minoritized (Latinx, Black, or Asian American). All caregivers identified as the LGBTQ youth’s biological mother. See Tables 1, 2, and 3 for participant characteristics.

**Table 1:** Youth Participant Characteristics

	Massachusetts (*n* = 7)		California (*n* = 9)		Total (*n* = 16)	
	Frequency (%)	Mean (SD)	Frequency (%)	Mean (SD)	Frequency (%)	Mean (SD)
Age (in years)		19.29 (0.95)		19.67 (1.41)		19.50 (1.21)
Sex assigned at birth (Female)	4 (57%)		8 (89%)		12 (75%)	
Gender identity						
Cis female	0 (0%)		4 (44%)		4 (25%)	
Cis male	3 (42%)		0 (0%)		3 (19%)	
Genderqueer/gender non-conforming	1 (14%)		0 (0%)		1 (6%)	
Non-binary	0 (0%)		2 (22%)		2 (13%)	
Other	0 (0%)		2 (22%)		2 (13%)	
Transgender male	3 (43%)		1 (11%)		4 (25%)	
Sexual orientation						
Bisexual/pansexual	2 (29%)		6 (67%)		8 (50%)	
Gay	1 (14%)		1 (11%)		2 (13%)	
Lesbian	0 (0%)		1 (11%)		1 (6%)	
Queer	0 (0%)		1 (11%)		1 (6%)	
Not reported	4 (57%)		0 (0%)		4 (25%)	
Race						
Asian	2 (29%)		0 (0%)		2 (13%)	
Black or African American	3 (43%)		0 (0%)		3 (19%)	
Other	0 (0%)		3 (33%)		3 (19%)	
White	2 (29%)		6 (67%)		8 (50%)	
Ethnicity (Latinx)	3 (43%)		9 (100%)		12 (75%)	
Highest level of education						
Less than high school	0 (0%)		1 (11%)		1 (6%)	
High school	7 (100%)		0 (0%)		7 (44%)	
Some college	0 (0%)		6 (67%)		6 (38%)	
Bachelor’s degree	0 (0%)		2 (22%)		2 (13%)	
Preferred language						
English	6 (86%)		8 (88%)		14 (88%)	
Spanish	0 (0%)		1 (11%)		1 (6%)	
Vietnamese	1 (14%)		0 (0%)		1 (6%)	
Interview language (English)	7 (100%)		9 (100%)		16 (100%)

**Table 2:** Caregiver participant characteristics

	Massachusetts (*n* = 5)	California (*n* = 6)	Total (*n* = 11)
	Frequency (%)	Frequency (%)	Frequency (%)
Caregiver’s highest level of education			
Less than high school	0 (0%)	1 (17%)	1 (9%)
High school diploma	1 (20%)	1 (17%)	2 (18%)
Some college	1 (20%)	1 (17%)	2 (18%)
Bachelor’s degree	2 (40%)	2 (33%)	4 (36%)
Graduate degree	1 (20%)	1 (17%)	2 (18%)
Caregiver’s Race			
Black or African American	3 (60%)	0 (0%)	3 (27%)
More than one race	1 (20%)	0 (0%)	1 (9%)
Other	0 (0%)	3 (50%)	3 (27%)
White	1 (20%)	2 (33%)	3 (27%)
Caregiver’s Ethnicity (Latinx)	2 (40%)	5 (83%)	7 (64%)
Caregiver’s preferred language			
English	3 (60%)	3 (50%)	6 (55%)
Spanish	1 (20%)	3 (50%)	4 (36%)
Portuguese	1 (20%)	0 (0%)	1 (9%)
Interview language (English)	4 (80%)	5 (83%)	9 (82%)
Child’s gender identity			
Cis female	0 (0%)	1 (17%)	1 (9%)
Cis male	1 (20%)	1 (17%)	2 (18%)
Genderqueer/gender non-conforming	1 (20%)	0 (0%)	1 (9%)
Non-binary	0 (0%)	2 (33%)	2 (18%)
Transgender female	1 (20%)	0 (0%)	1 (9%)
Transgender male	2 (40%)	2 (33%)	4 (36%)
Child’s sexual orientation			
Bisexual/pansexual	1 (20%)	4 (67%)	5 (46%)
Gay	0 (0%)	1 (17%)	1 (9%)
Lesbian	1 (20%)	0 (0%)	1 (9%)
Queer	0 (0%)	1 (17%)	1 (9%)
Not reported	3 (60%)	0 (0%)	3 (27%)

**Table 3:** CHW participant characteristics

	Massachusetts (*n* = 8)		California (*n* = 7)			Total (*n* = 15)	
	Frequency (%)	Mean (SD)	Frequency (%)	Mean (SD)		Frequency (%)	Mean (SD)
Age (in years)		37.88 (10.48)		51.43 (11.84)			44.20 (12.81)
CHW experience (in years)		6.50 (6.02)		9.71 (5.31)			8.00 (5.74)
Race							
American Indian/Alaska Native	1 (13%)		0 (0%)		1 (7%)		
Black or African American	2 (25%)		0 (0%)		2 (13%)		
More than one race	1 (13%)		0 (0%)		1 (7%)		
Other	1 (13%)		1 (14%)		2 (13%)		
White	3 (38%)		6 (86%)		9 (60%)		
Ethnicity (Latinx)	5 (63%)		7 (100%)		12 (80%)		
Highest level of education							
Less than high school	0 (0%)		1 (14%)		1 (6.7%)		
High school	0 (0%)		2 (29%)		2 (13%)		
Some college	1 (13%)		2 (29%)		3 (20%)		
Associate's degree	1 (13%)		1 (14%)		2 (13%)		
Bachelor’s degree	5 (63%)		0 (0%)		5 (33%)		
Some graduate work	1 (13%)		0 (0%)		1 (7%)		
Graduate degree	0 (0%)		1 (14%)		1 (7%)		
Interview language (English)	8 (100%)		0 (0%)		8 (53%)		

### Procedures

Youth and caregiver participants were recruited from medical centers in an urban area in Massachusetts and non-profit agencies in the central coast of California serving LGBTQ individuals, to have representation of all participant types from medical and community settings. CHWs included family navigators recruited from medical centers in Massachusetts and *promotoras* from community volunteer networks in California. Youth and caregiver participants were referred to the study through primary care physicians within medical centers in Massachusetts and were recruited in California using flyers advertised through community organizations that serve LGBTQ individuals. Recruitment emails were sent to medical centers in Massachusetts and community networks in California to recruit CHWs. All participants self-selected to be in the study. Researchers for the study had established community partnerships with all recruitment, but no previous relationships with participants. All participants completed an electronic survey that included demographic information and a 40–60-min semi-structured interview with a researcher trained in appropriate interviewing procedures, effective follow-up, and sensitivity to participant emotional reactions. The interviews were conducted between October 2020 and July 2021 in the participant’s preferred language (Spanish or English), with bilingual researchers conducting the interviews in Spanish. Participants received a $50 incentive for the interview. All interviews were audio-recorded and transcribed verbatim. All study procedures were deemed exempt by [masked] Institutional Review Boards.

Purposeful sampling was used with an effort to have variation across race, ethnicity, language spoken, and youth gender identity and sexual orientation. As recommended for qualitative research in implementation research, this variation was used to find shared and different patterns across cases (Palinkas et al., 2015). The research team met biweekly to discuss interview content and emerging themes. Recruitment continued until interviewers and non-interviewing team members noted saturation in themes. It has been found that meaning saturation, the point at which both themes have been identified and depth of understanding has been conveyed, occurs between 9 and 17 interviews (Hennink & Kaiser, 2022).

### Measures

#### Participant Characteristics

Youth participants were asked to describe their gender identity, sexual orientation, race, and ethnicity on categorical scales that allowed for more than one category to be selected and/or describe their own identity in a text field (Table 1). Caregivers were asked to describe the gender identity, sexual orientation, race, and ethnicity of their LGBTQ child. Caregivers were also asked to describe their own race, ethnicity, education level, and preferred language (Table 2). CHWs provided similar demographic information (Table 3) and information about their CHW experiences (e.g., years providing services, populations served).

#### Semi-Structured Interview

Three semi-structured parallel interview protocols were developed to guide the conversations with the participants, with a particular emphasis on cultural and linguistic influences. The interview guide was informed by the FRAME, with an emphasis on understanding why and how to adapt CHW models to the cultural needs of LGBTQ youth of color and their families. Before introducing the concept of working with CHWs, questions were asked about their experiences within their communities. For example, all stakeholders were asked about challenges (e.g., For youth, “*What are some challenges you have had to face or are currently facing as a [sexual orientation and/or gender identity] youth?;*” For caregivers, “*What are some challenges you have had to face or are currently facing as a parent of an LGBTQ youth*?”; For CHWs, “*What are some challenges that you have had to face or are currently facing working with parents of LGBTQ youth*?”). These questions were asked to understand areas to target within CHW intervention. After these broad questions, the definition and role of CHWs was introduced and follow-up questions were provided regarding perceptions of this role. Emphasis was placed on how CHW models should be adapted specifically in regards to (1) who should serve as CHWs (e.g., “*What characteristics should a CHW have when working with LGBTQ youth and families?”*), (2) CHW training *(e.g., “What specific training should this person receive?” “What should supervision look like for CHWs?”),* (3) and content that CHWs should be able to address (e.g., “*What specific resources as a [LGBTQ youth, parent of LGBTQ youth] would be helpful?”*). Broadly, CHW-specific questions explored their experiences working with LGBTQ youth and their caregivers and the types of training they received or desired to support this population (Table 4).

**Table 4:** Illustrative quotes of categories of themes from participants

	Participant group		
	CHW	Youth	Caregiver
Select Themes			
*Why are adaptations needed for LGBTQ youth and their caregivers?*			
*Theme: Experiences with stigma and discrimination often intersect with culture and religion*	“Absolutely, if your parents are more religious and depending on their culture, it can be more difficult for them to accept or even understand, not necessarily accept but mostly understand what you’re going through”	“a lot of times what I've noticed is people using Christianity as a way of excluding certain groups. For our specific conversation the LGBTQ community”	“So Mexicans, we're very close to well sometimes not even close to the family, but we really have this idea about family, about being close. You worry about what people say and what people think”
Who should serve as CHWs providing care?			
* Theme: Preference towards “relatable” providers that understand the cultural group they are serving*	“And if that person had an idea of that person's culture or beliefs, ways of life … you just get it without asking the zillion questions that I'm not ready to face right now, you just get it. You're just not judging this thought process because you are familiar with it. You can get straight to the roots of things without having to explore over and Over that process because you know exactly the things that trigger of it, in regards to where we're from. Therefore, I think they feel less judged.”	“…I am always comfortable with somebody who is maybe Latinx or like, LGBTQ, I think that’s something I-, you know, we can relate to. And being part of the community, sexuality, and so forth.”	“Language, I think, is very important, knowing the language, having the understanding… I think they should be Latino or Latina, Latinx. I think Spanish. Knowing the language and knowing the culture too is very important. I think the age also… just opening up to somebody younger makes it harder because we're supposed to know these things.”
How CHWs should be trained?			
*Theme: General LGBTQ education*	“Yeah, I think really just having a LGBTQ 101. These are the different—just to say that this is at the spectrum of what different families are experiencing.” “…I will like to have more information about even the history. Why not, a small brief description what is it? What are their struggles? Something pointed out general ideas, problematic that the group—the community have been dealing with and also to– not internalize but in order to actually get into the set of understanding more and also definitely now having the names it's not names, how they call themselves.”	“I feel like these folks should know the basics and also a little bit beyond. They don't have to be fluent in gender theory, but at least know more than just—know more, I feel." “Maybe have had experience—I don't want to say being a therapist but knowing how to carry a conversation, knowing how to address sensitive topics and—I feel like those are really important. How else should they be trained? Just in general, knowing a lot about the community, history so that they have a full understanding of everything.”	“Everything about LGBTQ… and also if they got a little exposure to current events and stuff on social media, too. Anything that's trending when it comes to that because I know my daughter's always looking at stuff when it comes to—they have challenges and things like that that's interesting to LGBT community. That's a good start but mainly anything that's going to help them as far as just the pronouns. I'm telling you, little things like that, that is serious.”
What content needs to be included in the intervention?			
*Theme: Provide education to caregivers*	[When asked about resources and services needed for caregiver of LGBTQ youth] “Well, first of all, education or information… talking to [the caregiver] and, uh, little by little they are opening their mindset of saying, 'Okay, this is the reality, and this is what I have to deal with.” [translated] “Bueno, primero que nada, educación o información.. el hablar con ellos y el que, eh, ellos poco a poco vayan abriendo su mentalidad en decir, 'Okay, la realidad es ésta, y esto es lo que tengo que enfrentar'.” [original]	“…I remember when it came to coming out and explaining things to my family, but you know I remember my mom wishing that she had someone to talk to… To be able to help her you know process and just like understand and learn… it was just so different and shocking for her where she was just basically felt like on her own. Having to understand something that she had no idea about. And I wish there was a way to be able to give her that support, because I feel like she tells me sometimes she's like you know, if I spoke English, it would be easier for me to go to like groups where we could talk, where I could talk about what it's like to have a child who's queer… I wish my mom especially had like a group that she could talk to, or just like resources that she could rely on to learn more that were in her language, and the language that she could understand."	“What is very difficult for me and that I never understand is like the pronouns… because for that I think that, if one has to study and know a lot about… that group because there are many things that we, at least, as caregivers or as Hispanics we don't understand…” [translated] “Lo que se me hace a mi muy dificil y que nunca entiendo es como los pronombres… porque para eso yo creo que, si tiene uno que estudiar y que saber mucho de… ese grupo porque son muchas cosas que nosotros, al menos, como padres o como Hispanos no entendemos…” [original]
*Theme: Link families to culturally appropriate services*	“I think it's because it depends, again, if you are an immigrant and you're not familiar with what is offered here in the United States, you are less—you're not going to find services quickly. It's not just for this topic but it's in general, I will say. It's much harder… I think culturally families are not aware of the services that can be out there because of the language barrier or just the idea what they come from or what they think.”	“I know there are LGBTQ support groups for people who speak English, but my caregivers do not speak English. And I feel for Vietnamese people, there is still a stigma, there’s not really much of a group or a program that supports them or they can go to.”	[When asked about resources they would have liked to have] “Having a community that looked like me and like spoke Spanish and had– I don't know, went to church. Yeah, just having that that community that'd be open about it. Yeah, having resources in Spanish, having the information in Spanish, having groups of people that I could just sit with and talk to about whatever was going on and just share our experiences."

### Data Analytic Plan

#### Qualitative Data Analysis

The current study utilized rapid qualitative assessment (RQA) to identify common themes that emerged from the interviews (Hamilton, 2013). RQA is a cost- and time-efficient approach to conducting qualitative analyses and has been shown to generate similar findings when compared to in-depth and thematic approaches to qualitative analysis (Gale et al., 2019; Taylor et al., 2018). RQA is a pragmatic, yet rigorous, approach that is a strong fit for time-sensitive research questions to identify and address real-world challenges in health care systems, such as understanding facilitators or barriers of delivering interventions (Lewinski et al., 2021). The present study sought to explore areas of adaptation for CHW models of care that supported LGBTQ youth and families. An RQA was conducted to inform appropriate and timely modifications to current CHW models (Lewinski et al., 2021), with the goal of implementing and evaluating these adapted models of care utilizing additional methodologies in clinical and community settings.

Data analysis was overseen by the entire authorship team and undergraduate research assistants. To conduct the RQA, the research team developed a summary template with domains corresponding with questions from the youth, caregiver, and CHW interview guides. Eight undergraduate and graduate level researchers were trained to extract key summary points relating to each domain from the transcripts (e.g., challenges, CHW training, resources needed), which were then entered into a matrix. The researchers completed a practice transcript to establish consistency in summaries and met throughout the course of the study to avoid drift in the way interviews were summarized. RQA is an especially valuable approach to compare responses from different participant groups to identify where themes converge and diverge (Leykum et al., 2022). The matrix approach allowed for comparison across different types of interviewees (youth, caregiver, CHW), locations (Massachusetts and California), CHW type (volunteer or hired navigator), and demographic characteristics. Every member of the research team reviewed the matrix and made notes of themes that they identified. These notes were discussed with the entire research group to identify and refine themes. The research group organized these themes under categories guided by the FRAME regarding the type of provider, CHW training, content of adaptations, and rationale for adaptations. After developing these themes, the interview transcripts were reviewed again to identify illustrative quotes for each theme. As needed, further refinement occurred through group discussion and consensus making.

The analysis team included members of the LGBTQ community, straight and cisgender individuals, caregivers and siblings of LGBTQ youth, Latinx individuals, and Asian American individuals. All interviews in Spanish were conducted and analyzed by native Spanish speakers. Disciplinary backgrounds included clinical psychology, nursing, public health, and pediatrics, with specific research expertise in implementation science, parenting interventions, CHW models, cultural adaptations, caregiver support for transgender youth, and LGBTQ youth mental health. Throughout the interview and analysis process the research team engaged in discussions about potential biases and assumptions that might arise as a result of their own lived experiences or lack thereof. These reflections and discussions led to the refinement of themes to help maximize objectivity in the preparation of the manuscript.

## Results

Qualitative themes were organized under categories informed by the FRAME in regards to adapting CHW-models for LGBTQ youth. The categories of themes addressed the following areas: (1) **Why** adaptations are needed for LGBTQ youth and their caregivers (i.e., goals for adaptations), (2) **Who** should serve as CHWs providing care (i.e., contextual adaptations related to personnel), (3) **How** CHWs should be trained (i.e., modifications to training), and (4) **What** content needs to be included in the intervention (i.e., modifications to content). The themes (italicized and bolded) within categories focused on similarities and differences relating to the experiences and challenges within and across stakeholder groups.

### Why Adaptations are Needed for LGBTQ Youth and Their Caregivers?

A primary theme was the ***lack of CHW training and experiences related to working with LGBTQ youth and their families***, and even those with some experience communicated difficulty providing support due to a lack of training related to the LGBTQ community (e.g., fear of misgendering). When recalling one experience working with an LGBTQ youth, a CHW expressed, *“…I found myself not knowing what pronouns to use… feeling uncomfortable in the situation because I didn’t want to offend her.”* Despite having limited experiences, the CHWs expressed ***a desire to provide support to working with LGBTQ youth and their families*** (“*… I have made it a goal to participate in webinars or sometimes the pediatric department has presentation with all staff and providers and would sometimes even invite families.*”); however, as one CHW explained “*good intentions*” alone are not enough to qualify someone to work with LGBTQ youth*, “You could approach someone with all due respect but still disrespect them because of your own lack of knowledge.”* Similarly, youth and caregivers described the need for care models and providers that aligned with CHW-models (i.e., social proximity, bridge to services). When describing the ideal characteristics of service providers, a Latinx, bisexual, genderqueer youth expressed:

*“Something that I guess I can think of out of my head is that I would hope that they could know, I guess knowing that homophobia and sexism and misogyny is deeply entrenched into the community. And having that cultural background and awareness, I feel like would be really crucial in being an intervening type of thing.”*

Overall, CHW, caregiver, and youth participants identified that CHW services for LGBTQ youth would need to be responsive to how ***experiences with stigma and discrimination often intersect with culture and religion***. A CHW described, *“I think it's more just what is—from their culture, religion, and stigma because a caregiver has a lot influences on them in some ways of how they can express. They feel okay to talk or not, so I think that would be one of the challenges for the youth.”* A Latinx, lesbian, non-binary youth explained, *“…there is so much machismo in the community that does not allow me to be my full self*.” Additionally, youth and their families described being discriminated against due to their race and how racism intersected with heterosexism, and cissexism. As one mother of a Black trans-male youth said:

“*… like police, they identify him now as a Black male. And to me, that—I worry about that because he’s been pulled aside from the police before just walking with his friends and searched for no reason. So, I worry about that*."

Given that CHWs are community members and often share similar cultural and religious backgrounds to the individuals they serve, CHWs are uniquely well-positioned to build trust across youth and caregivers, while navigating and addressing these challenges. As one CHW noted:

*“We, the promotoras, have this gift because we are people, they see us... as a family, like we go, uhm, to go help them, support them so that's why they open up, that's why they express themselves, ask for help because they know that we are the same, that we have gone through the same thing, perhaps the same more at the beginning when they arrive in this new country, then they feel with that confidentiality that they can count on us and that knowing they telling us their problems, that we are going to have that support and that help for them." [translated]*

*“Nosotras, las promotoras, tenemos ese don porque somos personas, nos ven... como una familia, como que nosotros vamos en, ehm eh, a llegar a ayudarlas, poyarlas entonces ellos por eso se abren, por eso ellos se expresan, piden ayuda entonces porque, que ellos saben que nosotros somos iguales, que hemos atravesado lo mismo, quizás los mismos más al principio al llegar a este país nuevo, entonces ellos se sienten con esa confidencialidad de que pueden contar con nosotros y que sabiendo ellos contándonos a nosotros sus problemas, que nosotros vamos a tener ese apoyo y esa ayuda para ellos.” [original]*

Specifically, youth and caregiver participants identified how ***adaptations to family navigation might be especially relevant for transgender youth***, who face additional systemic challenges stemming from stigma and discrimination within school and healthcare systems. As one Latinx, transgender youth recalled, *“The violence is so prevalent that it’s fearing for our safety, you know. And when I was in high school, and my, uh, town, it was, like, predominately Latinx, Hispanic, and I did not say I was trans.”* Many caregiver and youth participants communicated that several school systems did not accept the youth’s identities (e.g., continued misgendering, refusal to use affirmed name) or provide accommodations (e.g., lack of gender inclusive bathrooms). In health systems, transgender youth described experiencing discrimination in the form of misgendering, but also experienced difficulty in getting access to gender affirming care (e.g., hormone therapy), which are critical to a transgender youth’s sense of self and identity. Youth and caregivers suggested that family navigation could help coordinate care across systems that impact the youth.

### Who Should Serve as CHWs?

Youth, caregivers, and CHWs agreed that CHWs providing support to LGBTQ youth and families should be knowledgeable about the LGBTQ community, share an understanding of the culture and language of the family receiving support, be open-minded and nonjudgmental, and be an active listener. Indeed, having “*relatable*” ***providers that understand the cultural group they are serving*** was an important characteristic shared across all groups: *“…I would say, I would be more connected with a person of color because we still have some share, struggling like, racism, or… classism”* [Asian, gay male youth].

There were differences in perspectives on what the ***gender or sexual identity of the CHW*** should be, based on whether the role was meant to work with the youth or caregiver. Accordingly, youth and caregivers described LGBTQ youth providers as “peer partners” that provide mentorship as the youth navigates the community and related difficulties. An Asian, gay male youth, for example, noted:

*“… it would be more, makes sense if [the CHW] identify in our LGBTQ plus communities, because it's just really hard for a straight person to actually sympathize with what I'm saying. That's how I feel because maybe they can listen to us, but they wouldn't have the whole idea about it. They wouldn't actually experience, living experience.”*

Particpants also expressed the need for caregivers of LGBTQ youth to receive CHW support from someone who could build trust with a caregiver, educate them about the LGBTQ community (e.g., language, experiences, struggles), and, preferably, share the same culture but was not necessarily a member of the LGBTQ community. As described by one CHW, *“You understand them more and what they are going through because it’s the same culture.”*

### How Should CHWs be Trained to Work with LGBTQ Youth?

***CHWs desired greater education*** with respect to the LGBTQ community. Only 5 of the 15 CHWs interviewed for this study reported having any experience working with LGBTQ youth and/or their families. Of those who did have some experience, they reported discomfort due to their lack of knowledge and experience with this population. One CHW, for example, noted, *“one time I found myself not knowing what pronouns to use… feeling uncomfortable in the situation because I didn’t want to offend her”*. Indeed, ***general LGBTQ education*** such as the use of pronouns, gender identity, and sexual identity were all identified by youth, caregiver, and CHW participants as necessary education for CHWs to receive when working with LGBTQ youth and/or families.

Transgender youth and caregivers of transgender youth specifically noted the importance of CHWs receiving additional education about the transition process to better provide youth and families with information, resources, and support. Caregivers of transgender youth were especially interested in receiving this education and support, as knowing what to expect and how to best support their children through the process was an important piece of the support they’d hope to receive from CHWs. One caregiver, for example, expressed:

*“For me it has been difficult because as a mother, if I had a daughter, I want to see my daughter. And suddenly, getting used to the fact that he is not just another girl, he is a boy; his appearance changed because he started his testosterone treatment, and seeing his physical changes, that was hard for me too.” [translated]*

*“Para mí ha sido difícil porque como madre, si yo tuve una hija, yo quiero ver a mi hija. Y de repente, acostumbrarme a que él no es una niña más, es un boy; su apariencia cambió porque él empezó su tratamiento de testosterona, y verle los cambios físicos, eso fue duro también para mí” [original].*

CHW education of the process can facilitate more effective support for transgender youth and families, especially with respect to the expectations and navigation of the transition process.

Importantly, ***training CHWs to build trust and an understanding between the CHW and youth*,** especially around confidentiality and mediation was expressed across CHW and youth participants. When asked to describe training needs, one CHW noted, *“I think just like going into the room, feeling comfortable that you’re not going to like offend or disclose any information that you shouldn’t.”* Relatedly, a non-binary, lesbian youth stated,

*“It's very common for youth to feel-- LGBTQ youth, specifically, to feel kind of afraid to get services when they know that their family may or may not be supportive… So I feel like, being open to that fact and trying really hard to establish good relationships with the youth before they immediately jump into ways that they're trying to help…safety has always been my main priority, and that's often why I don't reach out to certain resources or certain people.”*

CHWs expressed wanting to more effectively navigate this type of communication to promote trust and safety for the LGBTQ youth they serve.

### What Content Should CHWs Address?

Overall, youth, caregivers, and CHWs agreed that family navigation for LGBTQ youth should ***provide education to caregivers*** and be knowledgeable of and refer families to available resources (e.g., mental health, medical, legal, support groups). One caregiver of a non-binary, Latinx youth, for example, expressed her own struggle to understand LGBTQ terminology by noting:

*“And just trying to understand the differences between sexuality and gender identity like that, that is like completely new to me. So trying to understand that and explain that, not that I would have to, but even if it came up, I don’t even know how I would try to explain it. I don’t have the language or the understanding sometimes.”*

Indeed, education about gender identity and sexual orientation was identified as a need for caregivers. As one Latinx caregiver of a gay male youth shared, *“… I feel like I’m the one doing all the research… it’s probably out there, but…I don’t know where to begin.”* Youth expressed frustration with having to provide this education to their caregivers, family members, and other members of their community (e.g., “*…I’m educating people all the time about who I am. And it’s exhausting. It’s not something I want to do all the time.”*).

Although family navigators embedded within medical centers expressed more familiarity with the resources available for LGBTQ youth compared to *promotoras* within community agencies, all CHWs voiced a desire to learn more about the available resources and services. Specifically, it was important for CHWs to be able to ***link families to culturally appropriate services***, as many participants noted that services available for LGBTQ youth were often not culturally or linguistically appropriate. Specific to transgender youth, youth and caregiver participants suggested it would be helpful to receive information about navigating complicated aspects of transgender healthcare, such as details on medical insurance coverage, hormone treatment and surgical procedures, as well as support with the social transition.

Both caregivers and CHWs, especially family navigators, emphasized that CHWs should be aware of current LGBTQ issues, policies, and legal rights in order act as an ***advocate across systems*** (e.g., school, medical, legal) for the youth. As one caregiver of a transgender youth noted, CHW should be:

*“aware of the law as well, and… the law protects, um LGBTQ children especially… their privacy, rights, and all these things because it’s just so, it’s so difficult… I've known instances of teachers who, who actually say something about an, an LGBTQ kid in their class, in the classroom and they are actually outing them out and um... So... that's something that, that has to be addressed.”*

Moreover, CHWs, caregivers, and youth noted that family navigation for LGBTQ youth might need to help mediate caregiver-child relationships and facilitate effective communication within the family. As one CHW shared:

*“I think a lot of times, because we're connecting with the head of the family for the most part, we play more of the role of a bridge between the family and providers. But I think there could be like a three directional bridge or model if you will. It's being that strength conductor between the caregiver and the child, the child and the caregiver, the child and the provider...”*

## Discussion

This study highlighted the role CHWs could play in enhancing access and culturally appropriate care for LGBTQ youth of color and their families. However, based on qualitative responses across participants, it was clear further adaptations are needed to traditional CHW models of care (e.g., family navigation) to meet the unique needs faced by LGBTQ youth. Whereas, CHW models are able to address many concerns faced within communities of color, including lack of insurance, limited availability of linguistically appropriate providers, and distrust of medical systems (Barnett et al., 2018a), adaptations were identified related to the types of training CHWs need related to LGBTQ identities and how these identities intersect with cultural values, and interventions they could deliver related to rejection within the family system and systemic barriers faced by LGBTQ youth.

Interestingly, CHWs and caregivers identified similar areas of training and education they would like regarding understanding sexual orientations and gender identities. Youth participants identified that it would be helpful if their caregivers received this information from individuals other than themselves, as this placed additional stress and pressure on them. As such, one role for CHWs could be to provide psychoeducation to caregivers related to sexual orientations and gender identities, and how to provide support to youth (Olson et al., 2016; Simons et al., 2013). Notably, the training received by CHWs should address the depth of the challenges LGBTQ youth encounter, especially as they relate to discrimination within their own family systems and society. Given these nuances, significant consideration should be given to building relationships that promote trust, safety, and confidentiality. Moreover, caregivers and LGBTQ youth may benefit from working with CHWs with different identities. Whereas youth may value peer support from other members of the LGBTQ community, youth and caregiver participants identified how the CHWs working with caregivers should share similar cultural backgrounds. This coincides with previous findings of transitional age youth reporting racial/ethnic concordance as an important aspect of peer providers’ roles and experiences (Hiller-Venegas et al., 2022). Indeed, for caregivers that have more stigma towards the LBTQ community, working with a CHW that holds a sexual orientation or gender identity that is different than their own might create a barrier to rapport building. For this reason, different workforces may be necessary to work with youth and caregivers. It is likely to have a larger public health impact if a range of CHWs are trained to work with LGBTQ youth and their families, than expecting they will share all intersecting identities of the youth or caregiver they are serving (e.g., the CHW also needing to be a caregiver to a child with the same sexual orientation and gender identity as the family they are working with). Rather, it is critical that, in addition to leveraging some shared characteristics, CHWs are trained to work across identities within a given community to best address the complexity of their needs.

Related to the content of CHW interventions, findings suggest that family navigation or other models where CHWs serve as bridges to services would be helpful in navigating the complex systems of care impacting LGBTQ youth and their caregivers, including schools, medical care, and mental health services. Having strong support models is crucial given the difficulties that LGBTQ youth of color face when engaging in mental health services, including potential barriers to engagement related to factors of stigma surrounding sexual orientation, issues surrounding cultural attitudes, and lack of knowledge regarding services (Moore et al., 2020; Wagaman, 2014). These findings seemed especially relevant for transgender and non-binary youth, given their needs related to receiving gender affirming medical care and addressing gender within school systems. Caregiver and CHW qualitative responses also suggested that CHWs can serve a significant role in advocacy for LGBTQ youth. This is especially relevant given the anti-LGBTQ legislations that are being passed in the US, such as Florida’s, “Don’t Say Gay” law (Diaz, 2022), and bans on gender affirming medical care and participation in sports for trans youth (Chappell, 2022; The Associated Press, 2022a, 2022b). The current political climate and discriminatory rhetoric is widening the care gap and limiting LGBTQ youth support systems. Now, more than ever, it is critical to implement accessible care models that support and advocate for LGBTQ youth.

## Strengths and Limitations

The present study reached LGBTQ youth of color, a highly marginalized population. It further diversified exploration by including caregivers of LGBTQ youth as well as CHWs embedded within community volunteer networks and medical centers. However, even with the strength of reaching this diverse population, limitations should be considered when interpreting the findings of the current study. Participants self-selected to participate in the study, and it is likely that the caregivers and CHWs who agreed to be interviewed may be more supportive of LGBTQ youth than is typical. Notably, the current sample was recruited from communities within liberal states with relatively adequate resources and is, therefore, not representative of the perspectives of individuals within less liberal and less resourced areas. This is especially notable given that many states are passing policies, which specifically discriminate against LGBTQ youth and their caregivers. Youth and their caregivers in states where anti-LGBTQ legislation is being passed likely need different types of interventions based on what is not only available, but also legal in the localities where they live.

## Conclusion

In recent months, the U.S. has witnessed a wave of anti-LGBTQ legislations that are impacting school instruction (Diaz, 2022), banning trans youth from playing school sports (The Associated Press, 2022b), denying youth gender-affirming medical care (The Associated Press, 2022a), and investigating caregivers and doctors who provide gender-affirming care (Chappell, 2022). New research shows that two thirds of LGBTQ youth report that the current political climate has had a detrimental impact on their mental health (The Trevor Project, 2022), indicating the substantial and growing need for access to affirmative mental health care. Given these events and the increasing support for CHW models across the US, it is a critical time to better understand how CHW interventions can be adapted to serve this vulnerable population. Themes in the current study point to important future directions to enhance equity for the families of LGBTQ youth of color. CHW models that are already being employed to enhance equity in access for communities of color should be adapted to enhance the training of CHWs in issues related to sexual orientation and gender identity. Specifically, this workforce has insights and expertise related to how religion and culture may impact parenting, but would benefit on training on how to enhance parental support for LGBTQ youth and increase access to services that are affirming to gender identity and sexual orientation. This was especially noted in the desire that CHWs had to address challenges LGBTQ youth and their families have, but lack of confidence in doing so. As previously discussed, training across different cultures, gender-identities, and sexual orientations can help CHWs better meet the diverse needs of their communities. These adapted models should be tested for effectiveness in enhancing parental supportive behaviors, increasing access to mental health services, and ultimately improving mental health symptoms in LGBTQ youth of color.
